# A retrospective analysis of geriatric trauma patients: venous lactate is a better predictor of mortality than traditional vital signs

**DOI:** 10.1186/1757-7241-21-7

**Published:** 2013-02-14

**Authors:** Kristin M Salottolo, Charles W Mains, Patrick J Offner, Pamela W Bourg, David Bar-Or

**Affiliations:** 1 Trauma Research Department, St. Anthony Hospital, 11600 W. 2nd Place, Lakewood, CO 80228, USA; 2 Trauma Research Department, Swedish Medical Center, Englewood, CO 80113, USA; 3 Trauma Services Department, St. Anthony Hospital, Lakewood, CO 80228, USA; 4 Rocky Vista University, Aurora, CO, 80011, USA

**Keywords:** Geriatric, Occult hypoperfusion, Shock index, Circulatory hemodynamic instability

## Abstract

**Background:**

Traditional vital signs (TVS), including systolic blood pressure (SBP), heart rate (HR) and their composite, the shock index, may be poor prognostic indicators in geriatric trauma patients. The purpose of this study is to determine whether lactate predicts mortality better than TVS.

**Methods:**

We studied a large cohort of trauma patients age ≥ 65 years admitted to a level 1 trauma center from 2009-01-01 - 2011-12-31. We defined abnormal TVS as hypotension (SBP < 90 mm Hg) and/or tachycardia (HR > 120 beats/min), an elevated shock index as HR/SBP ≥ 1, an elevated venous lactate as ≥ 2.5 mM, and occult hypoperfusion as elevated lactate with normal TVS. The association between these variables and in-hospital mortality was compared using Chi-square tests and multivariate logistic regression.

**Results:**

There were 1987 geriatric trauma patients included, with an overall mortality of 4.23% and an incidence of occult hypoperfusion of 20.03%. After adjustment for GCS, ISS, and advanced age, venous lactate significantly predicted mortality (OR: 2.62, p < 0.001), whereas abnormal TVS (OR: 1.71, p = 0.21) and SI ≥ 1 (OR: 1.18, p = 0.78) did not. Mortality was significantly greater in patients with occult hypoperfusion compared to patients with no sign of circulatory hemodynamic instability (10.67% versus 3.67%, p < 0.001), which continued after adjustment (OR: 2.12, p = 0.01).

**Conclusions:**

Our findings demonstrate that occult hypoperfusion was exceedingly common in geriatric trauma patients, and was associated with a two-fold increased odds of mortality. Venous lactate should be measured for all geriatric trauma patients to improve the identification of hemodynamic instability and optimize resuscitative efforts.

## Background

Initial triage, trauma team activation, and early resuscitation of trauma patients is often directed by the presence of abnormal traditional vital signs (TVS) such as systolic blood pressure (SBP) and heart rate (HR), as these measures are physical signs of circulatory hemodynamic instability (cHI). The shock index (SI), a composite of TVS, may be a more sensitive and accurate predictor of hypoperfusion and early shock than its individual components [1,2]. However, these markers may not be a sign of cHI in all patients following traumatic injury [3].

Geriatric patients respond to trauma and shock differently than their younger counterparts due to the presence of comorbid conditions, decreased physiologic reserve, elasticity of the vascular system, and concomitant medication use. Decreased physiologic reserve reduces the ability to respond to injury or tolerate aggressive resuscitation [4], while polypharmacy can alter the hemodynamic response to shock and complicate the patient’s clinical picture [5]. As a result, the severity of injury and the response to resuscitative efforts are often underestimated because the absence of tachycardia and hypotension can be misleading [6,7]. Martin et al. demonstrated that current triage criteria using SBP and HR are inadequate when applied to elderly trauma patients [7], leading to frequent undertriage in the elderly with a high risk of death [8].

Hypoperfusion is associated with the failure to maintain capillary oxygen delivery, leading to anaerobiosis and lactate production in excess to its rate of metabolism. The amount of lactate produced is believed to correlate with the total oxygen debt, the magnitude of hypoperfusion, and the severity of shock. Venous lactate (VL) is an accessible, accurate, validated measurement, and the measurement of admission lactate level may facilitate the recognition of occult hypoperfusion (OH) and shock [9-11], as VL is a metabolic indicator of cHI.

Elevated lactate levels have been consistently shown as a marker of severe injury and mortality in trauma patients, particularly in patients older than 55 years [10-16]. Despite this knowledge, few activation and resuscitation guidelines include VL as a measure of cHI, rather focusing on TVS like SBP and HR [17-19]. We instituted a geriatric resuscitation protocol in 2009 that includes a VL measurement for all geriatric trauma patients upon presentation to the emergency department (ED), and resuscitation based on VL values [20]. Prior to this new geriatric protocol, activation and resuscitation at our institution was based on mechanism, physical findings, Glasgow Coma Scale (GCS) and abnormal TVS. With advances in healthcare resulting in longer and healthier lifestyles in the elderly [21], there is greater potential for traumatic injury. Identifying an optimal marker of cHI has implications for initial assessment and resuscitation protocols for this ever increasing geriatric trauma population. We hypothesized that VL would have better predictive ability for mortality than SBP, HR, or SI in geriatric trauma patients.

## Methods

### Patients and setting

**Figure 1:**
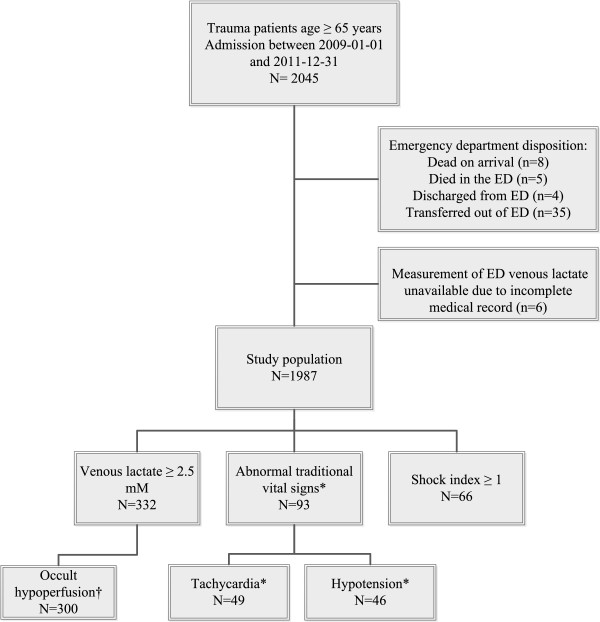
Patient disposition, by physiologic variable of interest. *Abnormal traditional vital signs: Hypotension (systolic blood pressure < 90 mm Hg) and/or tachycardia (heart rate > 120 beats/min). †Occult hypoperfusion: Venous lactate ≥ 2.5 mM with normal traditional vital signs.

This retrospective cohort study evaluated the medical records of all consecutively admitted geriatric (age ≥ 65) trauma patients from January 1st, 2009 through December 31st, 2011 to St. Anthony Hospital, an urban Level I Trauma Center located in Lakewood, Colorado. A trauma patient is defined according to a principal diagnosis of trauma based on ICD-9-CM diagnostic injury codes. Additional selection criteria can be found in the State registry manual [22]. Patients were excluded based on ED disposition, as follows: dead on arrival (n=8), died in the ED (n=5), discharged from the ED (n=4) or transferred-out of the ED (n=35, including transfers to pediatric trauma centers, burn centers, or Kaiser Permanente medical facilities), Figure 1. We also excluded patients for whom we were unable to determine if a VL had been drawn due to incomplete medical records (n=6). Dedicated trauma registrars prospectively abstracted data on all trauma admissions into our trauma registry (TraumaBase©, Conifer CO).

### cHI measurements

Vital signs, including SBP and HR measurements, were taken manually upon arrival to the ED, followed by automated measurements whose frequency was based on the patient’s acuity. After initial vital signs were measured, VL measurements were performed as follows: an anaerobic blood sample was obtained from an upper extremity peripheral venipuncture, or occasionally from a central line if one was placed emergently, and analyzed with the ABL-700 blood gas analyzer (Radiometer Analytical, Copenhagen Denmark). If the patient presented with an elevated VL, interventions included volume resuscitation and enhanced vigilance. If persistent, then central venous monitoring was performed if not already established.

### Outcome evaluation

The primary outcome was in-hospital mortality. The following variables were analyzed for their association with and predictive capability for in-hospital mortality: elevated VL (≥ 2.5 mM), hypotension (SBP < 90 mm Hg), tachycardia (elevated HR > 120 beats/minute), abnormal TVS (the presence of hypotension and/or tachycardia), and elevated shock index ≥ 1. An elevated VL in the ED ≥ 2.5 mM was used for this analysis based on previous studies that identified an increased risk of mortality with lactate ≥ 2.5 mM [23-25], and based on the geriatric resuscitation protocol’s VL parameters for initiating resuscitation protocols. We also examined mortality related to OH, defined as elevated VL in the presence of normal TVS.

Covariates included age, sex, ISS, GCS, mechanism of injury, respiratory rate, and admission service. These variables were categorized as follows: age (65–84 and ≥ 85 years), sex (male and female), ISS (≥ 16 and < 16), GCS (3–12 and 13–15), respiratory rate (< 10 or > 29 breaths/minute and 10–29), cause of injury (fall, motor vehicle accident, and all other types), and admission service (trauma service and all other services).

### Statistical analysis

Statistical analyses were performed using SAS® software, version 9.2 (SAS Institute, Cary, NC). We examined in-hospital mortality using Pearson and Fisher’s exact chi-square tests and multivariate logistic regression. Stepwise multivariate logistic regression models were used to examine whether the presence of the physiologic variables of interest (elevated VL, SI, HR, and SBP, abnormal TVS, and OH) independently predicted in-hospital mortality. All covariates that met entry and exit values of p < 0.15 were included in the stepwise logistic regression models, independent of whether they were significant predictors of mortality in univariate analyses. Each logistic regression model was built using separate stepwise selection; however, all models found the following variables to be significant at entry and exit values of p < 0.15: GCS, ISS and age. Adjusted R-squared and Hosmer-Lemoshow goodness of fit statistics were examined to assess how well the models fit the data; lower R-squared values and smaller p-values indicate a lack of fit of the model. Data are presented as odds ratios (OR) with 95% confidence intervals (CI), as well as medians with interquartile range. For all analyses, significance was set at p-value < 0.05. This study was approved by the St. Anthony Hospital Institutional Review Board.

## Results

### Patient demographics

There were 1987 geriatric trauma patients included in the study (Figure 1). The median age was 79 (72–86) years, and fall cause of injury occurred in 78.16% of patients. The most frequent service for admission was trauma surgery (55.11%), nonsurgical (e.g. critical care, medicine; 39.31%) and orthopedic services (4.78%). Overall injury severity was low with a median ISS of 9 (4–10) and median ED GCS of 15 (15–15). Despite the relatively minor injuries, the median length of stay was 4 (2–6) days, and the most common discharge disposition was to a skilled nursing facility (43.35%).

Ninety-three patients (4.72%) presented with abnormal TVS, and 66 patients (3.38%) presented with a SI ≥ 1, Figure 1. Of the 93 patients with abnormal TVS, 32 patients (39.02%) also had a known elevated VL. There were 1498 patients (75.39%) who had an ED VL drawn; of those, 332 patients (22.16%) had an elevated VL. Thirty-two patients (9.64%) with an elevated VL also presented with abnormal TVS; the remaining 300 patients (90.36%) presented with OH. Differences in clinical and demographic characteristics between patients with abnormal TVS and elevated VL are presented in Table 1.

**Table 1:** Demographic and clinical characteristics by presence of abnormal traditional vital signs (TVS) and elevated venous lactate*

**Variable, % (n)**	**Abnormal TVS (n=93)†**	**Lactate ≥ 2.5 mM (n=332)**	**missing (n)**
Abnormal TVS†	100 (93)	9.64 (32)	16
Lactate ≥ 2.5 mM	39.02 (32)	100 (332)	489
Mortality	13.98 (13)	12.05 (40)	0
Blunt mechanism	96.74 (89)	97.89 (325)	10
Female gender	49.46 (46)	48.49 (161)	0
Advanced age ≥ 85	20.43 (19)	25.30 (84)	0
ISS ≥ 16	38.89 (35)	27.58 (91)	29
GCS 3 - 8	22.58 (21)	15.06 (50)	27
Respiratory rate < 10 or > 29	6.82 (6)	3.79 (12)	62
Trauma team activation	53.76 (50)	40.66 (135)	0
Cause			0
Fall	65.59 (61)	64.16 (213)	
Motor vehicle	23.66 (22)	23.49 (78)	
ED disposition:			7
Intensive care unit	37.63 (35)	47.29 (157)	
Floor	31.18 (29)	35.24 (117)	
Discharge disposition:			1
LTAC/SNF/rehabilitation	65.59 (61)	46.69 (155)	
Home	17.20 (16)	33.73 (112)	

ISS, injury severity score; GCS, glasgow coma scale; ED, emergency department; LTAC, long term acute care; SNF, skilled nursing facility.

*There were 11 patients who had both abnormal TVS and elevated venous lactate.

†Abnormal TVS: Systolic blood pressure < 90 mm Hg and/or heart rate > 120 beats/min.

### Mortality

There were 84 patients who expired in the hospital (4.23%); approximately half of all deaths were patients with an elevated lactate (52.63%, 40/76 patients with a known lactate), while only 13 patients who expired presented with abnormal TVS (15.48%, 13/84).

As expected, mortality was significantly greater for patients with severe overall injuries (ISS ≥ 16 and < 16: 16.00% vs. 2.26%, respectively, p < 0.001), moderate to severe GCS (3–12 and 13–15: 36.97% vs. 2.12%, respectively, p < 0.001), and abnormal ED respiratory rate (< 10 or > 29 and 10–29: 24.00% vs. 3.79%, respectively, p < 0.001). Mortality was also significantly greater in males than females (5.51% and 3.15%, respectively, p=0.009), and admission to the trauma service than all other services (5.39% and 2.80%, respectively, p=0.004). There were no differences in mortality according to age (65–84 and ≥85: 3.84% and 5.18%, respectively, p=0.18), and cause of injury (3.73% falls, 5.45% motor vehicle accident, 6.78% all other causes, p=0.09).

Before adjustment, mortality was significantly different in patients with abnormal vs. normal TVS, SBP, HR, and SI (Table 2). However, after adjustment for ISS, GCS, and advanced age, mortality was not significantly higher in patients with abnormal TVS or SI ≥ 1 (Table 3), nor were the individual components significant: SBP < 90 mm Hg (OR: 1.93 [0.65 – 5.70], p = 0.24) and HR > 120 beats/min (OR: 1.41 [0.41 – 4.79], p = 0.58), data not shown.

**Table 2:** Comparison of in-hospital mortality rates, by presence of abnormal circulatory hemodynamic instability marker

	**Mortality % (n)**		
**Marker**	**Abnormal**	**Normal**	**P value**
Venous lactate	12.05 (40/332) *	3.09 (36/1166)	**< 0.001**
Traditional vital sign	13.98 (13/93) †	3.78 (71/1878)	**< 0.001**
Systolic blood pressure	19.57 (9/46) ‡	3.85 (74/1920)	**< 0.001**
Heart rate	12.24 (6/49) §	3.98 (76/1908)	**0.01**
Shock index	10.61 (7/66) ¶	3.82 (72/1884)	**0.02**

*Abnormal venous lactate: ≥ 2.5 mM.

†Abnormal TVS: Systolic blood pressure < 90 mm Hg and/or heart rate > 120 beats/min.

‡Abnormal systolic blood pressure: < 90 mm Hg.

§Abnormal heart rate: > 120 beats/min.

¶Abnormal shock index: ≥ 1.

**Table 3:** Stepwise multivariate logistic regression analysis: Predictors of in-hospital mortality

**Variable**	**Odds ratio**	**95% CI**	**P value**
Model 1: Lactate (r^2^=0.29; H-L=0.87)			
VL ≥ 2.5 mM	2.62	1.49 – 4.58	**< 0.001**
Glasgow coma scale 3-12	11.49	6.20 – 21.30	**< 0.001**
Injury severity score ≥ 16	3.40	1.86 – 6.21	**< 0.001**
Age ≥ 85 years	2.10	1.17 – 3.77	**0.01**
Model 2: Occult hypoperfusion* (r^2^=0.28; H-L=0.55)			
Occult hypoperfusion*	2.12	1.20 – 3.77	**0.01**
Glasgow coma scale 3-12	12.28	6.63 – 22.74	**< 0.001**
Injury severity score ≥ 16	3.47	1.90 – 6.36	**< 0.001**
Age ≥ 85 years	2.02	1.13 – 3.61	**0.02**
Model 2: TVS (r^2^=0.28; H-L=0.77)			
Abnormal TVS†	1.71	0.74 – 3.93	0.21
Glasgow coma scale 3-12	14.97	8.52 – 26.29	**< 0.001**
Injury severity score ≥ 16	3.88	2.23 - 6.76	**< 0.001**
Age ≥ 85 years	1.92	1.11 – 3.32	**0.02**
Model 3: Shock index (r^2^=0.27; H-L=0.69)			
Shock index ≥ 1	1.18	0.39 – 3.58	0.78
Glasgow coma scale 3-12	15.38	8.69 – 27.23	**< 0.001**
Injury severity score ≥ 16	3.86	2.19 – 6.81	**< 0.001**
Age ≥ 85 years	1.85	1.07 – 3.22	**0.03**
Model 4: Lactate and TVS (r^2^=0.29; H-L=0.72)			
VL ≥ 2.5 mM	2.58	1.47 – 4.52	**< 0.001**
Abnormal TVS†	1.46	0.62 - 3.47	0.39
Glasgow coma scale 3-12	11.23	6.06 – 20.83	**< 0.001**
Injury severity score ≥ 16	3.36	1.84 - 6.13	**< 0.001**
Age ≥ 85 years	2.14	1.19 – 3.84	**0.01**

Stepwise logistic regression, with entry and exit values set at p < 0.15 for all models.

Abbreviations: CI, confidence interval; VL, venous lactate; TVS, traditional vital signs; H-L, hosmer-lemeshow goodness of fit statistic.

*Occult hypoperfusion: VL ≥ 2.5 mM with normal TVS.

†Abnormal TVS: Systolic blood pressure < 90 mm Hg and/or heart rate > 120 beats/min.

Conversely, an elevated VL ≥ 2.5 mM with or without abnormal TVS was significantly associated with mortality both prior to and after adjustment for ISS, GCS, and advanced age (Table 3). There was a 2.62-fold increased odds of mortality in patients with a VL ≥ 2.5 mM, which persisted when patients with OH were analyzed.

When a multivariate regression incorporating both VL and TVS was performed, elevated VL continued to predict mortality (OR: 2.58, p < 0.001), whereas abnormal TVS was not predictive of mortality (OR: 1.46, p = 0.39), Table 3.

A moderate-to-severe GCS (3–12) was the most predictive of mortality in all four models, followed by overall injury severity (ISS ≥ 16); the best fitting model incorporated VL. Although the model’s estimates fit the data at an acceptable level according to the Hosmer-Lemoshow goodness of fit test, just 30% of the variability in mortality can be explained by the explanatory variables. The remaining 70% of the variability in mortality is explained by either inherent variability or variables not included in the models. Logistic regression models that included all variables with p < 0.15 in univariate analyses produced slightly lower R-squared values than the stepwise models presented in Table 3.

## Discussion

Guidelines for initial triage, trauma team activation, and early resuscitation following trauma are largely based on studies performed in younger trauma patients [26]. The geriatric population appears to be a relevant subset of patients in whom traditional physical signs of cHI such as SBP and HR may not identify severe injury and blood loss [6,7], and in whom additional indicators of cHI such as VL are important in predicting mortality. The results of this study demonstrate that a VL ≥ 2.5 mM was independently associated with a 2.6-fold increased odds of mortality in geriatric trauma patients. The TVS of SBP, HR, and SI were not independently associated with mortality in geriatric trauma patients, and activation based on abnormal TVS does not appear to independently identify a population of patients at risk of mortality. These results were demonstrated in a high volume level I trauma center with a large percentage of elderly trauma patients.

Perhaps more importantly, OH was exceedingly common in elderly trauma patients, occurring in 20.03%, and was associated with a two-fold increased odds of mortality. There continues to be a high frequency of OH ranging from 16% to 70% in hemodynamically stable severely injured patients [27-29]. At our institution we identified elderly trauma patients who appeared to be hemodynamically stable in the ED, and would later deteriorate in the operating room or in a non-intensive care unit setting because they were not adequately resuscitated or triaged. A protocol was implemented that utilizes VL on admission, and sets in motion a resuscitative protocol for patients with a VL ≥ 2.5 mM. It is important for inadequate perfusion to be recognized during initial resuscitation efforts, because prolonged OH in elderly trauma increases mortality from 12% to 35% [13]. A cut-off for VL of ≥ 2.5 mM was used for the geriatric resuscitation protocol based on existing literature [23-25]. A receiver operator characteristic curve analysis was performed in our population, and the results of this analysis support our definition of elevated VL: the optimal cut-off was found to be > 2.4 mM, with a sensitivity of 52.6% and a specificity of 79.5% for mortality.

Our results demonstrate that standard physiologic variables of SBP and HR may not identify increased risk of mortality in older trauma patients, while an elevated lactate can be used to identify patients with an increased risk for in-hospital mortality, signifying that an increased risk of mortality may not be recognized in current triage and resuscitation protocols that do not incorporate VL. The geriatric population may be especially susceptible to under-evaluation of risk because of comorbidities, polypharmacy and decreased physiologic reserve. We believe an initial VL drawn in the ED or in a pre-hospital setting may improve the identification of significantly injured geriatric patients. Further, we advocate the implementation of a geriatric-specific resuscitation protocol that includes an initial VL to aid in resuscitation and care protocols.

Several recent studies in non-geriatric trauma patients corroborate our findings that lactate is more strongly associated with mortality following trauma than standard physiologic variables [14,30,31]. Meregalli et al. examined 44 high-risk, hemodynamically stable trauma patients, and showed that nonsurvivors had higher lactate than survivors at 12 hours, 24 hours, and 48 hours, whereas there were no differences in mean arterial pressure, HR, and arterial blood oxygenation at any time between survivors and nonsurvivors [31]. On receiver operator characteristic curve analysis, the area under the curve was greater for lactate than for mean arterial pressure, HR, arterial bicarbonate, PaO_2_/FiO_2_, and urine output for predicting mortality. Vandromme et al. studied trauma patients in whom hemodynamic measures were indeterminate (SBP between 90–110 mm Hg), and demonstrated that lactate was a significantly better predictor of mortality than pre-hospital SBP and ED SBP [14]. Lavery and colleagues compared VL ≥ 2 mM against standard triage criteria in their ability to identify major injury, and showed that VL was significantly better in predicting ISS, intensive care unit resource utilization, and hospital length of stay [30].

Additionally, two studies in elderly trauma patients have shown that standard physiologic variables may not identify severe injury [3,6]: Lehmann et al. studied nearly 14000 geriatric trauma patients, and showed that HR and SBP were not predictive of severe injury [6], while Zarzuar and colleagues demonstrated that only in geriatric patients was age x SI a better predictor of mortality than HR, SBP, and SI alone [3].

A recent systematic review reported that initial lactate is useful for risk assessment, and an elevated lactate requires close monitoring for signs of deterioration [15]; yet few guidelines incorporate an initial lactate measurement [17-19]. Based on the results of this study, VL measurements should be used in the ED for patients for whom no activation occurs based on TVS. Further, we recommend that future studies examine the value of adding a point-of-care measurement of VL for either pre-hospital activation or delayed activation in the ED. While both venous and arterial lactate measurements have been shown to be markers of serious injury following trauma, VL eliminates the need for arterial sampling and correlates strongly with arterial lactate [15,30].

There are several potential limitations to our study: First, the study evaluated mortality in our geriatric trauma population, who sustained predominantly minor blunt injuries from fall and motor vehicle mechanisms of injury, which limits the ability to generalize our findings for trauma populations unlike our own. However, we believe the majority of elderly trauma patients seen at other institutions present with similar injury characteristics [12]. Second, compliance with VL measurements was 75.39%. Patients without a VL closely resembled patients with a VL < 2.5 mM for mortality and probability of survival; therefore, we believe the association between elevated VL and mortality would remain had all patients received a VL measurement. Third, due to the retrospective design we do not have all potentially relevant data to compare our studies to the existing literature, including emergent interventions in the ED, pre-injury location (e.g. home, nursing facility) and withdrawal of support. Fourth, there are additional activation and resuscitation parameters that we did not compare VL with, such as revised trauma score, GCS, and respiratory rate. Our study was intended to compare predictive ability of physical signs of cHI (SBP and HR) and metabolic indicators of cHI (VL) for mortality. Other institutions use base deficit as a metabolic marker of cHI; we did not examine base deficit because our geriatric resuscitation protocol specifies routine collection of VL.

## Conclusions

These results add to the growing body of literature demonstrating that VL is a predictor of mortality following trauma, and that standard physiologic variables of SBP and HR are not independent predictors of mortality in older trauma patients. These findings were demonstrated in a high volume level I trauma center with a large percentage of elderly trauma patients. Determining initial VL levels may improve the identification of cHI, resuscitative efforts, and outcome of geriatric patients, particularly those with unrecognized hypoperfusion.

## Abbreviations

TVS: Traditional vital signs; SBP: Systolic blood pressure; HR: Heart rate; CHI: Circulatory hemodynamic instability; SI: Shock index; VL: Venous lactate; OH: Occult hypoperfusion; ED: Emergency department; GCS: Glasgow coma score; ISS: Injury severity score; OR: Odds ratio; CI: Confidence interval.

## Competing interest

The authors declare that they have no competing interests.

## Authors’ information

St. Anthony Hospital, Trauma Research Department, Denver, CO (Mrs. Kristin Salottolo and Dr. David Bar-Or); St. Anthony Hospital, Trauma Services Department, Denver, CO (Drs. Mains and Offner and Mrs. Pamela Bourg); Swedish Medical Center, Trauma Research Department, Englewood, CO (Mrs. Kristin Salottolo and Dr. David Bar-Or); Rocky Vista University, Parker, CO (Drs. Bar-Or and Mains).

## Authors’ contributions

KS and DBO are responsible for the integrity of the data in this study and the accuracy of the data analysis. KS, PO and DBO were involved in writing the manuscript. KS and PB were involved in data selection and data collection. KS and PO conceived the study idea. CM and PB reviewed the manuscript for accuracy. All authors read and approved the final manuscript.

## Financial support

Support provided by St. Anthony Hospital, Lakewood, CO.

All the authors state that they have no financial or other conflicts of interest to disclose.

This study was presented as a poster presentation at the 2011 Annual meeting for the American Association for the Surgery of Trauma (AAST) in Chicago, IL.

## References

[B1] Allgower M Burri C ["Shock index"] Dtsch Med Wochenschr 1967 92 1947 1950 10.1055/s-0028-1106070 5299769

[B2] Paladino L Subramanian RA Nabors S Sinert R The utility of shock index in differentiating major from minor injury Eur J Emerg Med 2011 18 94 98 10.1097/MEJ.0b013e32833f212b 20842040

[B3] Zarzaur BL Croce MA Magnotti LJ Fabian TC Identifying life-threatening shock in the older injured patient: an analysis of the National Trauma Data Bank J Trauma 2010 68 1134 1138 10.1097/TA.0b013e3181d87488 20453769

[B4] Scalea TM Simon HM Duncan AO Atweh NA Sclafani SJ Phillips TF Shaftan GW Geriatric blunt multiple trauma: improved survival with early invasive monitoring J Trauma 1990 30 129 134 discussion 134–126 2304107

[B5] Victorino GP Chong TJ Pal JD Trauma in the elderly patient Arch Surg 2003 138 1093 1098 10.1001/archsurg.138.10.1093 14557126

[B6] Lehmann R Beekley A Casey L Salim A Martin M The impact of advanced age on trauma triage decisions and outcomes: a statewide analysis Am J Surg 2009 197 571 574 discussion 574–575 10.1016/j.amjsurg.2008.12.037 19393350

[B7] Martin JT Alkhoury F O'Connor JA Kyriakides TC Bonadies JA 'Normal' vital signs belie occult hypoperfusion in geriatric trauma patients Am Surg 2010 76 65 69 20135942

[B8] Rogers A Rogers F Bradburn E Krasne M Lee J Wu D Edavettal M Horst M Old and undertriaged: a lethal combination Am Surg 2012 78 711 715 22643270 10.1177/000313481207800628

[B9] Eastridge BJ Salinas J McManus JG Blackburn L Bugler EM Cooke WH Convertino VA Wade CE Holcomb JB Hypotension begins at 110 mm Hg: redefining "hypotension" with data J Trauma 2007 63 291 297 discussion 297–299 10.1097/TA.0b013e31809ed924 17693826

[B10] Jansen TC van Bommel J Mulder PG Rommes JH Schieveld SJ Bakker J The prognostic value of blood lactate levels relative to that of vital signs in the pre-hospital setting: a pilot study Crit Care 2008 12 R160 10.1186/cc7159 19091118 PMC2646325

[B11] Paladino L Sinert R Wallace D Anderson T Yadav K Zehtabchi S The utility of base deficit and arterial lactate in differentiating major from minor injury in trauma patients with normal vital signs Resuscitation 2008 77 363 368 10.1016/j.resuscitation.2008.01.022 18367305

[B12] Pudelek B Geriatric trauma: special needs for a special population AACN Clin Issues 2002 13 61 72 10.1097/00044067-200202000-00007 11852724

[B13] Schulman AM Claridge JA Young JS Young versus old: factors affecting mortality after blunt traumatic injury Am Surg 2002 68 942 947 discussion 947–948 12455785

[B14] Vandromme MJ Griffin RL Weinberg JA Rue LW 3rd Kerby JD Lactate is a better predictor than systolic blood pressure for determining blood requirement and mortality: could prehospital measures improve trauma triage? J Am Coll Surg 2010 210 861 867 867–869 10.1016/j.jamcollsurg.2010.01.012 20421067

[B15] Kruse O Grunnet N Barfod C Blood lactate as a predictor for in-hospital mortality in patients admitted acutely to hospital: a systematic review Scand J Trauma Resusc Emerg Med 2011 19 74 10.1186/1757-7241-19-74 22202128 PMC3292838

[B16] Neville AL Nemtsev D Manasrah R Bricker SD Putnam BA Mortality risk stratification in elderly trauma patients based on initial arterial lactate and base deficit levels Am Surg 2011 77 1337 1341 22127083

[B17] Antonelli M Levy M Andrews PJ Chastre J Hudson LD Manthous C Meduri GU Moreno RP Putensen C Stewart T Torres A Hemodynamic monitoring in shock and implications for management. International Consensus Conference, Paris, France, 27–28 April 2006 Intensive Care Med 2007 33 575 590 10.1007/s00134-007-0531-4 17285286

[B18] Bradburn E Rogers FB Krasne M Rogers A Horst MA Belan MJ Miller JA High-risk geriatric protocol: Improving mortality in the elderly J Trauma Acute Care Surg 2012 73 435 440 10.1097/TA.0b013e31825c7cf4 22846952

[B19] Jacobs DG Plaisier BR Barie PS Hammond JS Holevar MR Sinclair KE Scalea TM Wahl W Practice management guidelines for geriatric trauma: the EAST Practice Management Guidelines Work Group J Trauma 2003 54 391 416 10.1097/01.TA.0000042015.54022.BE 12579072

[B20] Bourg P Richey M Salottolo K Mains CW Development of a geriatric resuscitation protocol, utilization compliance, and outcomes J Trauma Nurs 2012 19 50 56 22415508 10.1097/JTN.0b013e31822b80f5

[B21] Federal Interagency Forum on Aging Related Statistics Older Americans 2000: Key Indicators of Well-Being 2000 National Center for Health Statistics http://www.agingstats.gov/Main_Site/Data/2000_Documents/entire_report.pdf. Access date: January 3, 2013

[B22] Emergency Medical and Trauma Services Section Colorado Trauma Registry Inclusion/Exclusion Criteria 2011 Colorado Department of Public Health and Environment http://www.colorado.gov/cs/Satellite?blobcol=urldata&blobheadername1=Content-Disposition&blobheadername2=Content-Type&blobheadervalue1=inline%3B+filename%3D%22Registry+Manual+Section+A+-+Inclusion+Exclusion.pdf%22&blobheadervalue2=application%2Fpdf&blobkey=id&blobtable=MungoBlobs&blobwhere=1251848990095&ssbinary=true. Access date: January 3, 2013

[B23] Callaway DW Shapiro NI Donnino MW Baker C Rosen CL Serum lactate and base deficit as predictors of mortality in normotensive elderly blunt trauma patients J Trauma 2009 66 1040 1044 10.1097/TA.0b013e3181895e9e 19359912

[B24] Howell MD Donnino M Clardy P Talmor D Shapiro NI Occult hypoperfusion and mortality in patients with suspected infection Intensive Care Med 2007 33 1892 1899 10.1007/s00134-007-0680-5 17618418

[B25] Shapiro NI Howell MD Talmor D Nathanson LA Lisbon A Wolfe RE Weiss JW Serum lactate as a predictor of mortality in emergency department patients with infection Ann Emerg Med 2005 45 524 528 10.1016/j.annemergmed.2004.12.006 15855951

[B26] Phillips S Rond PC 3rd Kelly SM Swartz PD The failure of triage criteria to identify geriatric patients with trauma: results from the Florida Trauma Triage Study J Trauma 1996 40 278 283 10.1097/00005373-199602000-00018 8637079

[B27] Blow O Magliore L Claridge JA Butler K Young JS The golden hour and the silver day: detection and correction of occult hypoperfusion within 24 hours improves outcome from major trauma J Trauma 1999 47 964 969 10.1097/00005373-199911000-00028 10568731

[B28] Claridge JA Crabtree TD Pelletier SJ Butler K Sawyer RG Young JS Persistent occult hypoperfusion is associated with a significant increase in infection rate and mortality in major trauma patients J Trauma 2000 48 8 14 discussion 14–15 10.1097/00005373-200001000-00003 10647559

[B29] Thom O Taylor DM Wolfe RE Myles P Krum H Wolfe R Pilot study of the prevalence, outcomes and detection of occult hypoperfusion in trauma patients Emerg Med J 2010 27 470 472 10.1136/emj.2009.073254 20360482

[B30] Lavery RF Livingston DH Tortella BJ Sambol JT Slomovitz BM Siegel JH The utility of venous lactate to triage injured patients in the trauma center J Am Coll Surg 2000 190 656 664 10.1016/S1072-7515(00)00271-4 10873000

[B31] Meregalli A Oliveira RP Friedman G Occult hypoperfusion is associated with increased mortality in hemodynamically stable, high-risk, surgical patients Crit Care 2004 8 R60 R65 10.1186/cc2423 15025779 PMC420024

